# Circulating tumour cells to drive the use of neoadjuvant chemotherapy in patients with muscle-invasive bladder cancer

**DOI:** 10.1016/j.esmoop.2022.100416

**Published:** 2022-03-03

**Authors:** N. Beije, I.E. de Kruijff, J.J. de Jong, S.O. Klaver, P. de Vries, R.A.L. Jacobs, D.M. Somford, E. te Slaa, A.G. van der Heijden, J. Alfred Witjes, L.M.C.L. Fossion, E.R. Boevé, J. van der Hoeven, H.H.E. van Melick, C.J. Wijburg, H. Bickerstaffe, J.W.M. Martens, R. de Wit, J. Kraan, S. Sleijfer, J.L. Boormans

**Affiliations:** 1 Department of Medical Oncology and Cancer Genomics Netherlands, Erasmus MC Cancer Institute, Erasmus University Medical Center, Rotterdam, The Netherlands; 2 Department of Urology, Erasmus MC Cancer Institute, Erasmus University Medical Center, Rotterdam, The Netherlands; 3 Department of Urology, Maasstad Hospital, Rotterdam, The Netherlands; 4 Department of Urology, Zuyderland Medical Center, Heerlen, The Netherlands; 5 Department of Urology, Canisius-Wilhelmina Hospital, Nijmegen, The Netherlands; 6 Department of Urology, Isala Hospital, Zwolle, The Netherlands; 7 Department of Urology, Radboud University Medical Center, Nijmegen, The Netherlands; 8 Department of Urology, Maxima MC, Eindhoven, The Netherlands; 9 Department of Urology, Franciscus Gasthuis & Vlietland, Rotterdam, The Netherlands; 10 Department of Urology, Reinier de Graaf Gasthuis, Delft, The Netherlands; 11 Department of Urology, St. Antonius Hospital, Nieuwegein, The Netherlands; 12 Department of Urology, Rijnstate Hospital, Arnhem, The Netherlands; 13 Department of Urology, Bravis Hospital, Roosendaal, The Netherlands

**Keywords:** circulating tumour cells, neoadjuvant chemotherapy, muscle-invasive bladder cancer, CTCs, liquid biopsy

## Abstract

**Background:**

Guidelines recommend neoadjuvant chemotherapy (NAC) for the treatment of nonmetastatic muscle-invasive bladder cancer (MIBC). NAC is, however, underutilized in practice because of its associated limited overall survival (OS) benefit and significant treatment-related toxicity. We hypothesized that the absence of circulating tumour cells (CTCs) identifies MIBC patients with such a favourable prognosis that NAC may be withheld.

**Patients and methods:**

The *CirGuidance* study was an open-label, multicentre trial that included patients with clinical stage T2-T4aN0-N1M0 MIBC, scheduled for radical cystectomy. CTC-negative patients (no CTCs detectable using the CELLSEARCH system) underwent radical surgery without NAC; CTC-positive patients (≥1 detectable CTCs) were advised to receive NAC, followed by radical surgery. The primary endpoint was the 2-year OS in the CTC-negative group with a prespecified criterion for trial success of ≥75% (95% confidence interval (CI) ±5%).

**Results:**

A total of 273 patients were enrolled. Median age was 69 years; median follow-up was 36 months. The primary endpoint of 2-year OS in the CTC-negative group was 69.5% (*N* = 203; 95% CI 62.6%-75.5%). Two-year OS was 58.2% in the CTC-positive group (*N* = 70; 95% CI 45.5%-68.9%). CTC-positive patients had a higher rate of cancer-related mortality [hazard ratio (HR) 1.61, 95% CI 1.05-2.45, *P* = 0.03] and disease relapse (HR 1.87, 95% CI 1.28-2.73, *P* = 0.001) than CTC-negative patients. Explorative analyses suggested that CTC-positive patients who had received NAC (*n* = 22) survived longer than CTC-positive patients who had not (*n* = 48).

**Conclusion:**

The absence of CTCs in MIBC patients was associated with improved cancer-related mortality and a lower risk of disease relapse after cystectomy; however, their absence alone does not justify to withhold NAC. Exploratory analyses suggested that CTC-positive MIBC patients might derive more benefit from NAC.

**Trial registration:**

Netherlands Trial Register NL3954; https://www.trialregister.nl/trial/3954

## Highlights

• Two-year OS in CTC-negative MIBC patients did not meet the criterion for trial success.
• CTCs are prognostic for cancer-related mortality and relapse-free survival in MIBC.
• CTC-positive MIBC patients survived longer when receiving NAC in explorative analyses.
• The absence of CTCs alone is insufficient to withhold NAC in MIBC patients.

## Introduction

Radical cystectomy with pelvic lymph node dissection and urinary diversion is the mainstay of treatment for patients with nonmetastatic muscle-invasive bladder cancer (MIBC). Despite this extensive surgery, MIBC patients have a poor 5-year overall survival (OS) of 57.9%.^1^ This poor outcome is mainly attributable to the development of distant metastases. Neoadjuvant chemotherapy (NAC) as a means to eliminate micrometastases in MIBC patients has been extensively studied and there is level 1a evidence that NAC leads to an absolute OS benefit of 5%-6% at 5-10 years.^2^^,^^3^ Therefore, international guidelines recommend cisplatin-based NAC in MIBC patients who are fit for cisplatin chemotherapy according to Galsky’s criteria.^4^, ^5^, ^6^ Nevertheless, NAC is underutilized in clinical practice,^7^, ^8^, ^9^ which may be due to physicians’ reluctance to prescribe NAC to elderly patients with comorbidities, the delay in time to radical surgery and the relatively modest benefit of NAC.^10^ Thus, there is an unmet clinical need for biomarkers to determine whether an MIBC patient will or will not benefit from NAC, guiding physicians and patients in the treatment decision-making process. A minimally invasive biomarker in cancer patients is the use of circulating tumour cells (CTCs), which are tumour cells that circulate in the peripheral blood of patients with solid malignancies. The prognostic value of CTCs, enumerated with the CELLSEARCH system, has been shown for various epithelial malignancies in the metastatic and the nonmetastatic settings,^11^ including MIBC.^12^, ^13^, ^14^, ^15^ Given the superior survival in MIBC patients without CTCs compared with patients with CTCs,^12^^,^^13^ we hypothesized that the absence of CTCs could identify a subgroup of MIBC patients with such a good prognosis that NAC can be withheld. To investigate this hypothesis, we initiated the *CirGuidance* study in patients with clinical stage T2-T4aN0-1M0 MIBC.^15^ Patients without detectable CTCs at the moment of MIBC diagnosis received radical cystectomy without NAC and encompassed the primary study population. If one or more CTCs were detected, the patient was advised to undergo NAC followed by radical cystectomy.

## Patients and methods

### Patients and study design

The *CirGuidance* study was an open-label, multicentre, biomarker-driven trial in 15 hospitals in The Netherlands. Eligible patients were aged ≥18 years and were required to have histopathologically confirmed muscle-invasive urothelial carcinoma of the bladder as assessed by diagnostic transurethral resection of the bladder tumour. The clinical stage had to be T2-T4aN0-1M0 as assessed by bimanual examination under general anaesthesia and by computed tomography of thorax and abdomen within 6 weeks prior to study registration. The treating physician must have had the intention to perform a radical cystectomy as local treatment. Exclusion criteria were the presence of >50% of nonurothelial variant histology (e.g. squamous, glandular or neuro-endocrine differentiation); history of another malignancy in the past 5 years; known or suspected prostate cancer and the intention to treat the patient with systemic adjuvant therapy after radical cystectomy. Of note, adjuvant chemotherapy is not routinely applied in The Netherlands, based on the lack of strong evidence supporting its associated OS benefit^16^ and its advantage over deferred chemotherapy.^17^

The study protocol was approved by the medical ethics review board of Erasmus MC, Rotterdam, The Netherlands (number 2013-301) and the ethics committees or institutional review boards of each participating centre. Written informed consent was obtained from all patients. The study was registered at The Netherlands Trial Register (NL3954).

### Procedures

The presence of CTCs was assessed in 10 ml of blood drawn in CellSave tubes and sent to a central laboratory (Erasmus MC, Rotterdam, The Netherlands), where it was processed within 96 h after the blood draw. CTCs were enumerated using the CELLSEARCH circulating tumour cell kit (Menarini Silicon Biosystems, Castel Maggiore, Italy). All CTC enumeration results were reviewed by two trained operators before being made final.

A patient’s treating physician received the result of the CTC enumeration within 1 week of the blood draw. For patients without CTCs present in 7.5 ml of blood (i.e. ‘CTC negative’), the protocol dictated immediate radical cystectomy without NAC. For patients having one or more CTCs detectable (i.e. ‘CTC positive’), the treating physician was advised to apply NAC before radical cystectomy, but could choose to withhold NAC, either at one’s own discretion, according to local guidelines, or in case of contraindications to cisplatin.

### Endpoints

The primary endpoint was the 2-year OS in CTC-negative patients, defined as the survival status 2 years following study registration. Secondary endpoints were OS, cancer-specific mortality (defined as the time from study registration until death that was definitely or probably attributable to bladder cancer, or intervention related) and the cumulative incidence of relapse (defined as the time from registration to local or metastatic relapse, whichever came first).

### Statistical analysis

The sample size calculation was based on the presumption that a 2-year OS of 75% in MIBC patients without detectable CTCs would be an OS not justifying NAC. The rationale for this presumption was that the additional benefit of NAC, associated with a hazard ratio (HR) of 0.86 in the Advanced Bladder Cancer (ABC) Collaboration meta-analysis,^2^ would result in an OS benefit from NAC of <5% if the 2-year OS was 75%. The cut-off for a significant OS benefit of 5% was chosen based on the Dutch national criteria (PASKWIL) for superiority of a (neo)adjuvant treatment, dictating that a neoadjuvant treatment should result in at least 5% OS benefit. To find a 2-year OS of 75% with a 95% confidence interval (CI) based on a precision of ±5%, 188 CTC-negative patients had to be included (reflecting an associated benefit of NAC of 2.9%-4.1% assuming an HR of 0.86 with NAC). Given an expected prevalence of detectable CTCs in 25% of the patients,^12^ 260 patients had to be screened. During the study, the number of ineligible patients was higher than expected, and therefore the study was amended to include 320 patients, which was approved by the Erasmus MC Medical Ethics Review Board.

All endpoints were assessed in the intention-to-treat population. The primary endpoint was assessed using the Kaplan–Meier method in CTC-negative patients for whom survival data were available 2 years after registration. All other endpoints were assessed in all patients. The median follow-up duration was defined as the median follow-up duration in patients alive at the date of last follow-up. Baseline clinical and histopathological characteristics were compared between groups using the Fisher exact test for discrete variables and the Mann–Whitney *U* test for continuous variables. OS was estimated using the Kaplan–Meier method, and differences between groups in survival analyses were assessed using the log-rank test. Cancer-specific mortality and the cumulative incidence of relapse were estimated by a Fine and Gray cumulative incidence function. For cancer-specific mortality, death from a cause other than bladder cancer was considered a competing risk; for relapse, death of any cause was a competing risk. Stata version 13.0 (StataCorp, College Station, TX) was used for the analyses. *P* values <0.05 were considered significant.

## Results

### Patient characteristics

**Figure 1:**
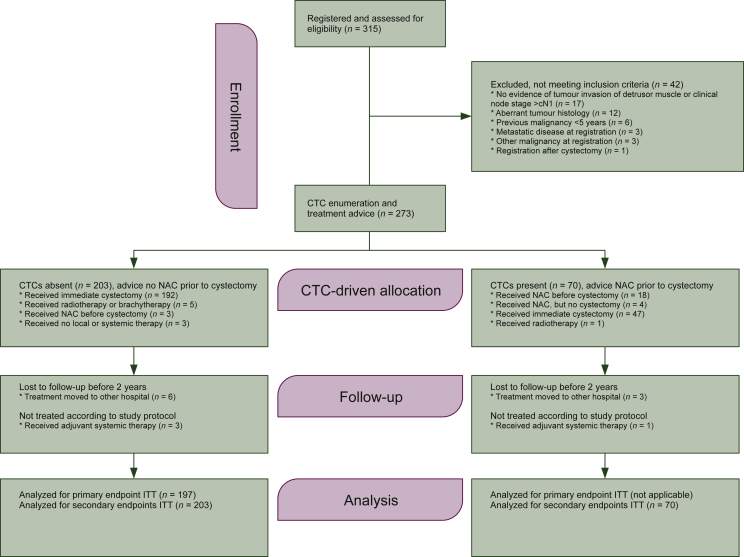
**Study diagram of the CirGuidance study, according to CONSORT guidelines.** CTC, circulating tumour cell; ITT, intent to treat; NAC, neoadjuvant chemotherapy.

A total of 315 patients had been assessed for study eligibility between 15 October 2013 and 5 April 2018 (Figure 1). Forty-two did not meet the inclusion criteria; thus CTC enumeration was performed and treatment advice was given for 273 patients. The CTC-negative group comprised 203 patients (74.4%) and these patients were per protocol required to undergo immediate radical cystectomy without NAC. The CTC-positive group comprised 70 patients (25.6%) with a median of 1 CTC/7.5 ml (interquartile range 1-3, range 1-26); it was advised to treat these patients with NAC followed by radical cystectomy.

Baseline characteristics of CTC-negative versus CTC-positive patients are described in Table 1. CTC-positive patients were older than CTC-negative patients with a median of 70.9 versus 68.5 years, respectively (*P* = 0.02) and had higher clinical tumour stages (*P* = 0.02), reflecting especially more clinical T4a stage (11 versus 3%, respectively). Two-year follow-up data were available for the vast majority of patients in the CTC-negative group (*n* = 197, 97%) and the CTC-positive group (*n* = 67, 96%). The median follow-up duration was 36 months. There was nonadherence to the protocol in 14 patients in the CTC-negative group: 11 patients did not receive radical cystectomy, while 3 received adjuvant chemotherapy after cystectomy. Despite the recommendation to treat CTC-positive patients with NAC, only 22 of the 70 CTC-positive patients (31.4%) received NAC. The most important reasons for not prescribing NAC were preference of the including hospital or physician (*n* = 20), comorbidity (*n* = 18) or patient refusal (*n* = 7; Supplementary Table S1, available at https://doi.org/10.1016/j.esmoop.2022.100416). Nonadherence to the protocol occurred in two CTC-positive patients: one patient received external radiotherapy to the bladder instead of surgery; another received adjuvant chemotherapy.

**Table 1:** Baseline characteristics of 273 patients with nonmetastatic muscle-invasive bladder cancer who were included in the *CirGuidance* study, according to CTC status

Characteristic	All patients (*n* = 273)	CTC-negative patients (*n* = 203)	CTC-positive patients (*n* = 70)	*P* value
Age (years), median (interquartile range)	69 (61-74)	68.5 (61-64)	70.9 (65-75)	0.02
Gender, *n* (%)				0.55
Male	190 (70)	139 (68)	51 (73)	
Female	83 (30)	64 (32)	19 (27)	
Previous NMIBC, *n* (%)				>0.99
No	220 (81)	163 (80)	57 (81)	
Yes	53 (19)	40 (20)	13 (19)	
Smoking status, *n* (%)				0.60
Never	59 (22)	43 (21)	16 (23)	
Currently smoking	113 (41)	87 (43)	26 (37)	
Past smoker	92 (34)	65 (32)	27 (39)	
Unknown	9 (3)	8 (4)	1 (1)	
Clinical tumour stage, *n* (%)				0.02
cT2	154 (56)	120 (59)	34 (49)	
cT3	105 (39)	77 (38)	28 (40)	
cT4a	14 (5)	6 (3)	8 (11)	
Clinical node stage, *n* (%)				0.76
cN0	259 (95)	193 (95)	66 (94)	
cN1	14 (5)	10 (5)	4 (6)	

*P* values represent comparisons between the CTC-negative group and the CTC-positive group.

CTC, circulating tumour cell; NMIBC, nonmuscle-invasive bladder cancer.

### Survival outcomes

**Figure 2:**
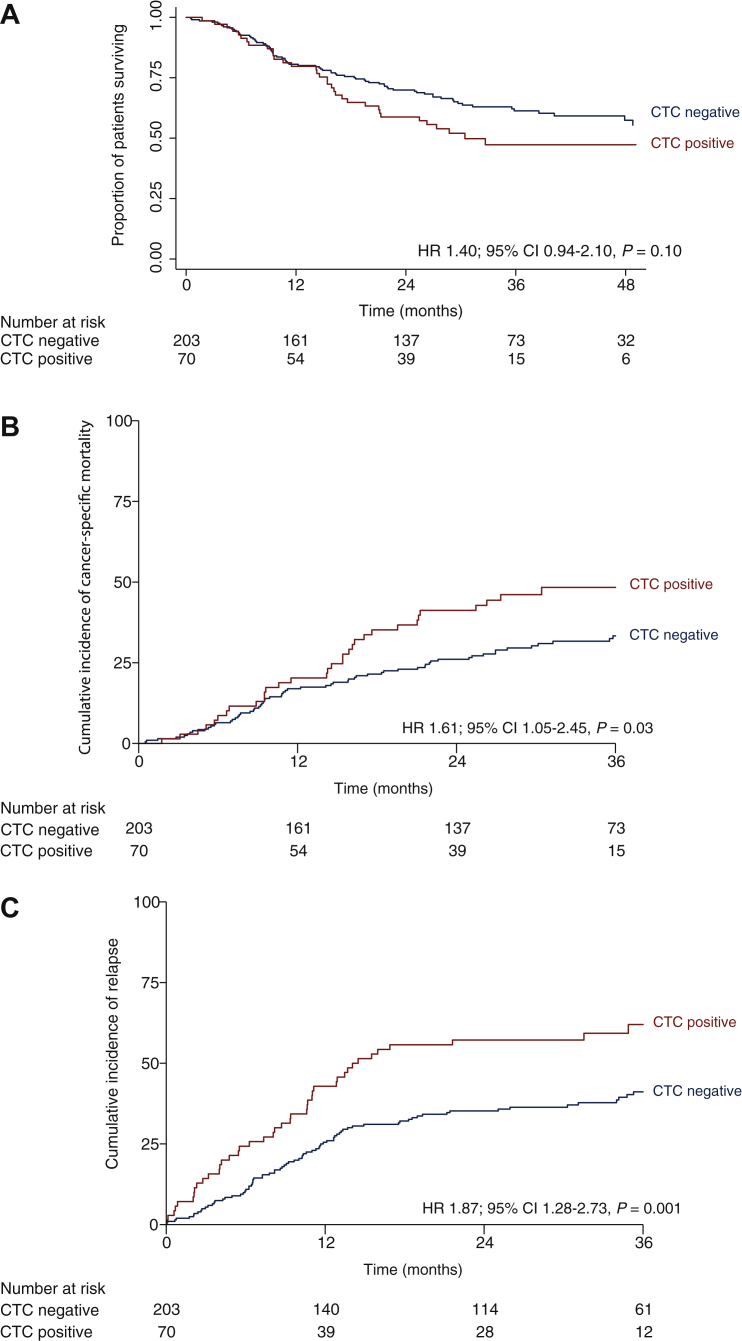
**Survival estimates of overall survival in 273 patients with clinical stage T2-T4aN0-1M0 muscle-invasive bladde cancer, stratified by circulating tumour cell (CTC) status.** (A) overall survival, (B) cumulative cancer-related mortality and (C) cumulative incidence of relapse. CI, confidence interval; HR, hazard ratio.

The primary endpoint of 2-year OS in CTC-negative patients was 69.5% (95% CI 62.6%-75.5%). The 2-year OS in CTC-positive patients was 58.2% (95% CI 45.5%-68.9%). OS did not statistically significantly differ between CTC-negative and CTC-positive patients (HR 1.40, 95% CI 0.94-2.10, *P* = 0.10; Figure 2A). Notably, 15 of 112 total deaths (13%) were noncancer related. The 2-year cancer-specific survival in CTC-negative patients was 73.9% (95% CI 67.0%-79.4%) and in CTC-positive patients it was 58.8% (95% CI 47.4%-70.6%). The cancer-specific survival in CTC-positive patients was significantly shorter than that in CTC-negative patients (HR 1.61, 95% CI 1.05-2.45, *P* = 0.03; Figure 2B). Disease relapses occurred significantly more in CTC-positive patients than in CTC-negative patients (HR 1.87, 95% CI 1.28-2.73, *P* = 0.001; Figure 2C).

### Pathological outcomes and response to NAC

Supplementary Table S2, available at https://doi.org/10.1016/j.esmoop.2022.100416 gives the pathological characteristics of the radical cystectomy and pelvic lymph node specimens for CTC-negative patients and CTC-positive patients who had not received NAC. The proportion of those with pT3-pT4 disease was 76% in CTC-positive patients who had not received NAC and had undergone a radical cystectomy (*n* = 47) versus 51% in CTC-negative patients who had undergone a radical cystectomy (*n* = 195; *P* = 0.004). The higher tumour stage in CTC-positive patients who had not received NAC was also reflected in higher proportions of positive surgical margins and tumours that were not surgically resectable. The pathological N-stage, the presence of lymphovascular invasion and the presence of concomitant prostate cancer did not differ significantly between the groups.

The most applied chemotherapeutic regimen for patients in the CTC-positive group who received NAC (*n* = 22) was gemcitabine and cisplatin (*n* = 20, 91%); one patient received gemcitabine and carboplatin, and one patient received methotrexate, vinblastine, adriamycin and cisplatin. All but one of these 22 patients received two or more cycles of NAC; in the exceptional case, NAC was stopped because of toxicity after one cycle. The pathological response was a complete response defined as the absence of residual cancer (ypT0N0) in six patients (27%), ypTisN0 in four (18%) and ≥ypT2 disease or ypTxN+ disease in eight (37%). Four patients (18%) did not undergo a radical cystectomy because of tumour progression during NAC.

### Outcomes in CTC-positive patients who received NAC

**Figure 3:**
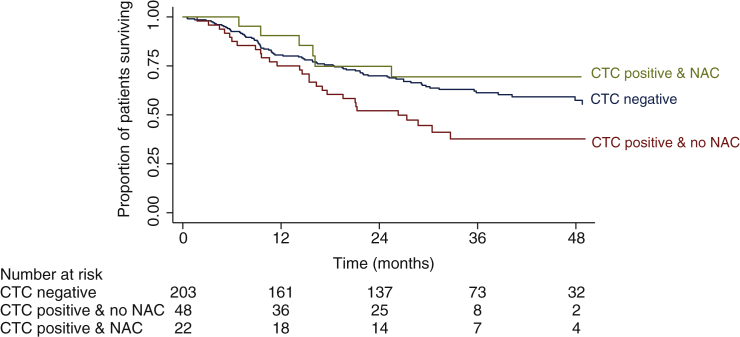
**Kaplan–Meier estimates of overall survival in 273 patients with clinical stage T2-T4aN0-1M0 muscle-invasive bladder cancer according to circulating tumour cell (CTC) status and whether or not neoadjuvant chemotherapy had been received in the CTC-positive patient group.** CTC, circulating tumour cell; NAC, neoadjuvant chemotherapy.

CTC-positive patients who had not received NAC were significantly older than CTC-positive patients who did receive NAC (median 72 versus 67 years; *P* = 0.005), while their clinical tumour stage did not differ significantly (Supplementary Table S3, available at https://doi.org/10.1016/j.esmoop.2022.100416). The impact of NAC on OS and the cumulative incidence of relapse was explored. The 2-year OS was 74.8% (95% CI 49.5%-88.8%) in CTC-positive patients who had received NAC versus 52.0% (95% CI 37.2%-65.0%) in CTC-positive patients who had not received NAC (Figure 3). The reason for not receiving NAC (i.e. local/physician preference or patient refusal versus other reasons) did not appear to influence the OS (Supplementary Figure S1, available at https://doi.org/10.1016/j.esmoop.2022.100416). Exploration of the cumulative incidence of relapse did not indicate that CTC-positive patients who had received NAC had fewer relapses than CTC-positive patients who had not received NAC (Supplementary Figure S2, available at https://doi.org/10.1016/j.esmoop.2022.100416).

## Discussion

The *CirGuidance* study aimed to establish the clinical utility of CTC detection as a prognostic marker in nonmetastatic MIBC patients scheduled to undergo radical cystectomy. Based on previous literature highlighting the prognostic value of CTCs in MIBC, we aimed to investigate whether NAC could safely be withheld in the subgroup of MIBC patients in whom no CTCs could be detected, as the added benefit of NAC in that subgroup with a favourable outcome would be too small. In this CTC-negative group, the 2-year OS after surgery without NAC was 69.5% (95% CI 62.6%-75.5%). Given the prespecified criterion of a 2-year OS of 75% (95% CI ±5%) justifiable to withhold NAC in CTC-negative patients, the 2-year OS and corresponding lower confidence interval in this study yielded insufficient evidence to withhold NAC in CTC-negative patients.

Patients in this study had a relatively advanced age, with a median age of 69 years, especially in comparison with the meta-analysis published by the ABC Collaboration,^2^ which reported a median of 63 years and on which the present study sample size calculation was based. The relatively advanced age of patients in our study may have contributed to the failure to meet the prespecified criterion for trial success, as we found a high rate of noncancer-specific mortality at 13%. Given these factors and taking into account the noncancer-related 2-year OS of 73.9% in the CTC-negative group, the probability of a significant survival benefit of NAC (i.e. of >5%) might still be small, irrespective of not meeting the prespecified criterion for trial success.

Although CTC-negative patients in this study had longer OS than CTC-positive patients, the difference was not statistically significant, which is in contrast to previous reports.^12^^,^^13^ While the present study was not powered to explore differences between CTC-negative versus CTC-positive patients, potential explanations could be the high noncancer-specific mortality rate in this study, or lessening of the OS differences as a consequence of subsequent treatments. Either of these explanations is plausible, given that the CTC-negative patients did have a lower cancer-specific mortality and fewer relapses than CTC-positive patients. The difference could also be explained by an improved survival in CTC-positive patients who had received NAC. Explorative analyses revealed that CTC-positive patients who had received NAC had a longer OS than CTC-positive patients who had not received NAC. It could be speculated, therefore, that NAC is especially useful in CTC-positive patients, as the presence of CTCs might reflect a higher probability of micrometastases being present at the moment of diagnosis, which could be eliminated by NAC. Patients receiving NAC may have had a better general condition than patients not receiving NAC, thus explaining the observed higher survival rate. However, explorative OS analyses taking into account the reason why patients did not receive NAC did not reveal clear differences between patients who did not receive NAC because NAC was contraindicated versus patients in whom NAC was not contraindicated (i.e. local or physician preference of patient refusal). Because the number of CTC-positive patients who had received NAC was small and the beneficial effect of NAC did not become apparent in the incidences of relapse, the observed effect might also be spurious.

Limitations of this study were the nonrandomized design and the fixed 2-year OS endpoint, which was derived from data from the ABC meta-analysis on the added value of NAC in MIBC. While this study was not designed or appropriately powered to analyse the CTC-positive population, the limited number of CTC-positive patients who received NAC limits the exploratory analyses in the CTC-positive population. The fact that most of these patients were not treated with NAC because of preference of the including hospital or physician further demonstrates the ongoing physician’s dilemma of benefit versus harm of NAC in MIBC patients. Even in the presence of a biomarker with known strong prognostic value, such as CTCs as used in this study, only 31.4% of the CTC-positive patients had received NAC, which is only slightly higher than the 21% of an unselected population of MIBC patients in a previous study in The Netherlands.^18^ Another limitation of the study is that the assay used to detect CTCs is dependent on the expression of the epithelial marker EpCAM, which may have led to undetected CTCs that have undergone epithelial–mesenchymal transition.^19^

### Conclusions

The *CirGuidance* study further demonstrated the prognostic value of CTCs for cancer-specific mortality and incidence of relapse in nonmetastatic MIBC patients treated by radical cystectomy. With a 2-year OS of 69.5% in CTC-negative patients, however, the prespecified criterion for trial success was not met, indicating that the absence of CTCs alone is insufficient to withhold NAC in MIBC patients. In addition, we found an indication of improved survival in CTC-positive patients who had received NAC versus those who had not received NAC. At best, CTC enumeration at the moment of diagnosis may serve as an additional criterion to clinical characteristics to guide the decision to prescribe NAC in MIBC patients.
