# Supplementary material for: Circulating tumour cells to drive the use of neoadjuvant chemotherapy in patients with muscle-invasive bladder cancer
Source: ESMO Open. 2022 Mar 3;7(2):100416. doi: 10.1016/j.esmoop.2022.100416 (PMC9058916; doi:10.1016/j.esmoop.2022.100416)
Supplement: Supplementary material [file mmc1.docx]

**SUPPLEMENTARY TABLES AND FIGURES**

| **Reason for not receiving NAC** | **Number of patients** |
| --- | --- |
| Preference of including hospital or physician | 20 |
| Comorbidity: renal | 10 |
| Comorbidity: impaired hearing | 3 |
| Comorbidity: peripheral vascular disease | 2 |
| Comorbidity: other | 3 |
| Patient refusal (but eligible patient) | 7 |
| Poor performance status | 2 |
| Recent infectious episode | 1 |

**Supplementary table 1.** Reasons for not receiving NAC.

| **Characteristic** | **CTC-negative patients**  **(n=195)** | **CTC-positive patients & no NAC (n=47)** | **P-value** |
| --- | --- | --- | --- |
| **Pathological tumor stage**  pT0-pT1  pT2  pT3  pT4 | 47 (24%)  48 (25%)  77 (39%)  23 (12%) | 7 (15%)  4 (9%)  23 (49%)  13 (27%) | 0.004 |
| **Pathological node stage**  N0  N1-3 | 145 (75%)  50 (26%) | 32 (68%)  15 (32%) | 0.46 |
| **Lymphovascular invasion**  No  Yes | 133 (68%) 62 (32%) | 27 (57%)  20 (43%) | 0.17 |
| **Surgical margins**  Microscopic radical (R0)  Microscopic irradical (R1)  Residual tumor (R2) | 184 (94%) 5 (3%) 6 (3%) | 39 (83%)  4 (8.5%)  4 (8.5%) | 0.03 |
| **Pathological prostate cancer**  Yes  No  Unknown | 100 (73%)  36 (26%) 1 (1%) | 28 (76%)  6 (16%)  3 (8%) | 0.38 |

**Supplementary table 2.** Post-cystectomy characteristics for all patients who have undergone cystectomy (or surgery was started, but aborted because of inoperable disease). The group of CTC-positive patients who received NAC is not included, details on pathological staging following NAC in this group are described in the text.

| **Characteristic** | **CTC-positive and no NAC (n=48)** | **CTC-positive and NAC (n=22)** | **P-value** |
| --- | --- | --- | --- |
| **Age, years**  Median (IQR) | 72 (67 – 76) | 67 (60 – 71) | 0.005 |
| **Clinical tumor stage**  cT2  cT3  cT4a | 25 (52%)  16 (33%)  7 (15%) | 9 (41%)  12 (54%)  1 (5%) | 0.20 |
| **Clinical node stage**  cN0  cN1 | 45 (94%)  3 (6%) | 21 (95%)  1 (5%) | 0.63 |

**Supplementary table 3.** Baseline characteristics of CTC-positive patients, according to whether or not they received NAC.


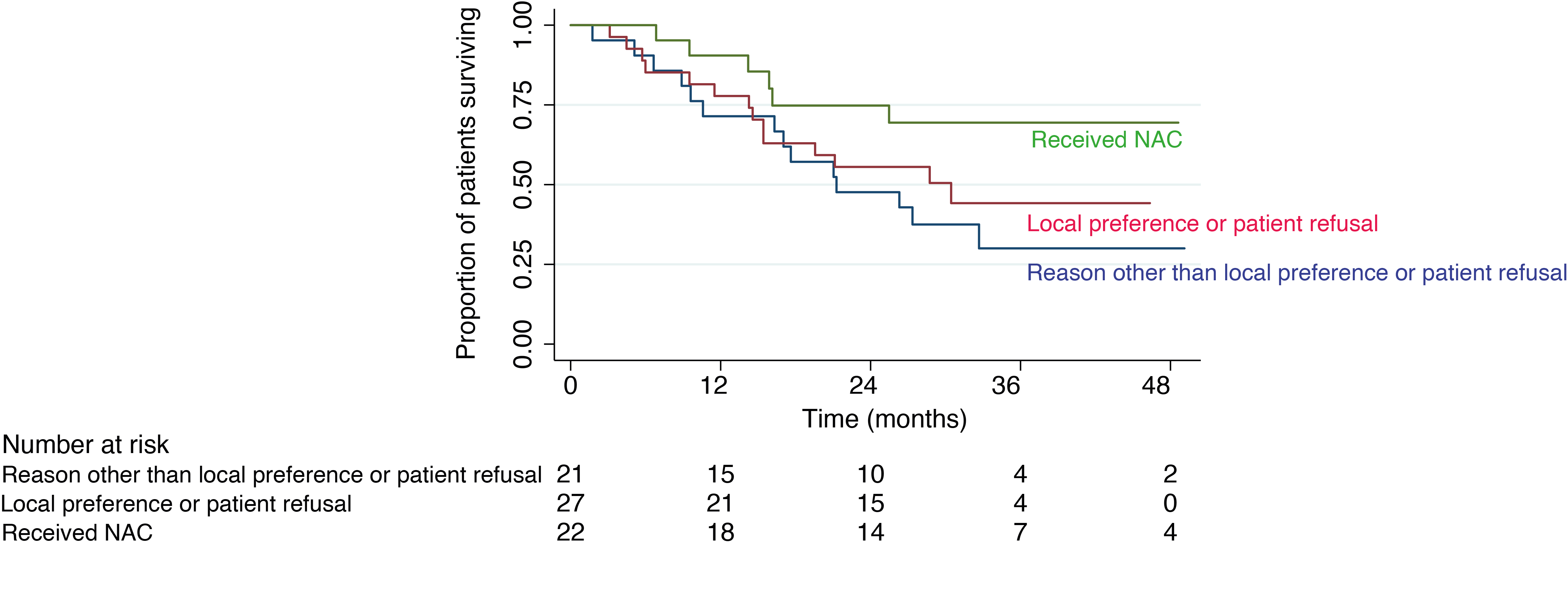


**Supplementary figure 1.** Kaplan-Meier estimates of overall survival according to whether or not the patient had received NAC and if not, the reason for not receiving NAC

**
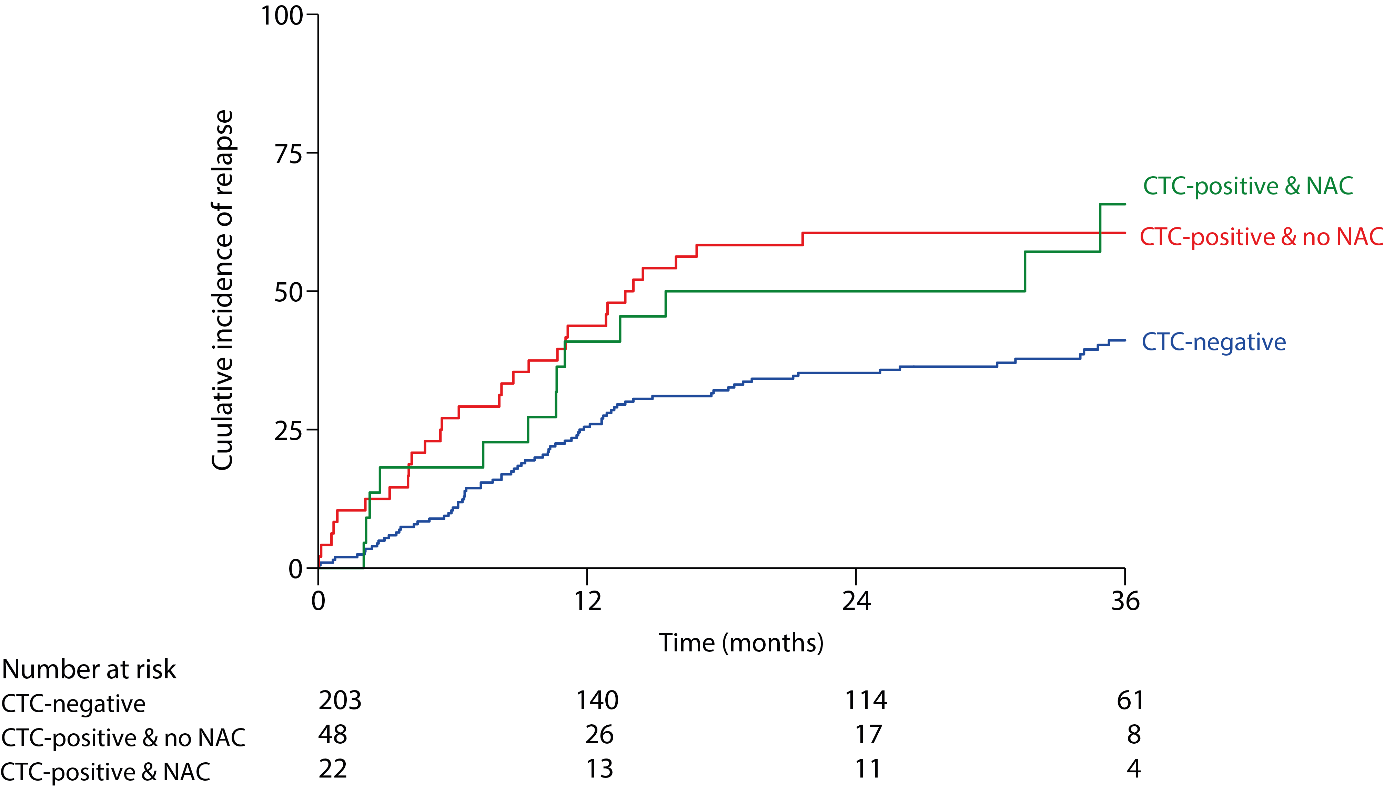
Supplementary figure 2.** Cumulative incidence of relapse according to CTC status and whether or not neoadjuvant chemotherapy had been received in the CTC-positive group
